# O Teste de Caminhada de 6 Minutos prevê a melhora física em longo prazo de sobreviventes à unidade de terapia intensiva: um estudo de coorte prospectiva

**DOI:** 10.5935/0103-507X.20210056

**Published:** 2021

**Authors:** Regis Goulart Rosa, Camila Dietrich, Enio Luiz Tschiedel do Valle, Denise Souza, Luciana Tagliari, Mariana Mattioni, Túlio Frederico Tonietto, Rosa da Rosa, Mirceli Goulart Barbosa, Gisele Agustini Lovatel, Pedro Dal Lago, Eubrando Silvestre Oliveira, Daniel Sganzerla, Juliana M. S. Andrade, Paula Berto, Paulo Ricardo Cardoso, Evelin Carneiro Sanchez, Maicon Falavigna, Juçara G. Maccari, Gabriela Rech, Caroline Robinson, Daniel Schneider, Patrícia de Leon, Lívia Biason, Cassiano Teixeira

**Affiliations:** 1 Hospital Moinhos de Vento - Porto Alegre (RS), Brasil.; 2 Universidade Federal de Ciências da Saúde de Porto Alegre - Porto Alegre (RS), Brasil.; 3 Hospital Ernesto Dornelles - Porto Alegre (RS), Brasil.; 4 Hospital de Clínicas de Porto Alegre, Universidade Federal do Rio Grande do Sul - Porto Alegre (RS), Brasil.

**Keywords:** Cuidados intensivos, Assistência ao convalescente, Reabilitação, Desempenho físico funcional, Teste de esforço

## Abstract

**Objetivo:**

Avaliar a capacidade do Teste de Caminhada de 6 Minutos para predizer a melhora do estado funcional físico em longo prazo de pacientes sobreviventes à unidade de terapia intensiva.

**Métodos:**

Foram avaliados, de forma prospectiva, entre fevereiro de 2017 e agosto de 2018, em um ambulatório pós-unidade de terapia intensiva, 32 sobreviventes à unidade de terapia intensiva. Foram inscritos consecutivamente os pacientes com permanência na unidade de terapia intensiva acima de 72 horas (para admissões emergenciais) ou acima de 120 horas (para admissões eletivas) que compareceram ao ambulatório pós-unidade de terapia intensiva 4 meses após receberem alta da unidade de terapia intensiva. A associação entre a distância percorrida no Teste de Caminhada de 6 Minutos realizado na avaliação inicial e a evolução do estado funcional físico foi avaliada durante 8 meses, com utilização do Índice de Barthel.

**Resultados:**

A distância média percorrida no Teste de Caminhada de 6 Minutos foi significantemente mais baixa nos sobreviventes à unidade de terapia intensiva do que na população geral (405m *versus* 557m; p < 0,001). A idade (β = -4,0; p < 0,001) e a fraqueza muscular (β = -99,7; p = 0,02) se associaram com a distância percorrida no Teste de Caminhada de 6 Minutos. A distância percorrida no Teste de Caminhada de 6 Minutos se associou com melhora do estado funcional físico no período de 8 meses de acompanhamento desses pacientes (razão de chance para cada 10m: 1,07; IC95% 1,01 - 1,16; p = 0,03). A área sob a curva Característica de Operação do Receptor para predição da melhora funcional física pelo Teste de Caminhada de 6 Minutos foi de 0,72 (IC95% 0,53 - 0,88).

**Conclusão:**

O Teste de Caminhada de 6 Minutos, realizado 4 meses após a alta da unidade de terapia intensiva, predisse com precisão moderada a melhora do estado funcional físico de sobreviventes à unidade de terapia intensiva.

## INTRODUCTION

The long-term physical, cognitive, and mental health disabilities that often affect survivors of critical illness are associated with decreased quality of life for subjects and their families.^(1,2)^ Additionally, physical impairment after discharge from the intensive care unit (ICU) complicates access to rehabilitation and return to work or studies and has been associated with death.^(3-5)^

The 6-Minute Walk Test (6MWT) is a standardized measure of functional exercise capacity^(6)^ that has been proposed as a prognostic factor for subjects with chronic disorders such as chronic obstructive pulmonary disease (COPD), heart failure, and pulmonary arterial hypertension.^(7-10)^ Among COPD subjects, for example, the distance covered during the 6MWT has been associated with hospitalizations and survival.^(7,8)^ The 6MWT is easy to perform, well tolerated, safe, and more reflective of activities for daily living than other walking tests.^(11,12)^ Moreover, the 6MWT is frequently used in cohorts of ICU survivors to assess recovery of exercise capacity;^(13-16)^ however, the literature evaluating the 6MWT as a predictor of long-term physical improvement in this population is scarce. Accordingly, the present study aimed to evaluate the 6MWT as a predictor of long-term physical improvement in general ICU survivors attending a post-ICU outpatient clinic.

## METHODS

This prospective cohort study was conducted with consecutive ICU survivors attending a post-ICU outpatient referral clinic that provides follow-up care to subjects from four tertiary hospitals in Porto Alegre (RS), Brazil. The 6MWT was performed at the clinic 4 months after discharge from the ICU as part of the baseline physical assessment of these subjects. Subjects were followed up for eight months using structured telephone interviews.

The study was conducted in accordance with good clinical practice and approved by the institutional review boards of all participating centers. Consent for participation was obtained from all study subjects or their proxies. The inclusion criteria were age ≥ 18 years, ICU stay ≥ 72 hours in cases of emergency medical or surgical admissions or ≥ 120 hours in cases of elective surgical admissions. The exclusion criteria were the presence of any contraindications or limitations to perform the 6MWT (i.e., inability to walk, unstable cardiac disease, unstable respiratory disease, severe cognitive impairment, or any medical contraindication).

### Definitions

Characteristics related to ICU stay (ICU admission type, risk of death at ICU admission, diagnosis of sepsis, organ dysfunction during ICU stay, and length of ICU stay) were obtained retrospectively through review of medical records by site investigators of each of the four hospitals referring subjects to the outpatient clinic. The risk of death at ICU admission was calculated according to the Acute Physiology and Chronic Health Evaluation II (APACHE II)^(17)^ or the Simplified Acute Physiology Score 3 (SAPS 3).^(18)^ Sepsis was defined according to sepsis 2 criteria.^(19)^ Organ dysfunction was defined as the presence of any of the following conditions during the ICU stay: need for invasive mechanical ventilation, vasopressors, renal replacement therapy (except for subjects under chronic dialysis treatment), parenteral nutrition, blood or blood product transfusion, and *delirium* (measured according the Confusion Assessment Method for the ICU).^(20)^

Variables related to the patient’s health status at the post-ICU outpatient evaluation - during the first 7 days after ICU discharge (age, educational attainment, comorbidities, physical functional status, muscle strength, frailty, cognitive status, resilience and symptoms of anxiety, depression, and posttraumatic stress disorder - PTSD) were evaluated by trained investigators during face-to-face assessments with validated tools. Comorbidities were assessed using the Charlson Comorbidity Index (CCI; scores range from zero to 33, with higher scores indicating greater comorbidity).^(21)^ The CCI score was dichotomized into low (zero or one) or high comorbidity (≥ 2). Physical functional status was assessed using the Barthel Index (BI; scores range from zero to one hundred, with higher scores indicating better functional status).^(22)^ Subjects were classified as independent (BI > 95), mildly dependent (BI > 75 to 95), moderately dependent (BI > 50 to 75), or severely dependent (BI ≤ 50). Muscle strength was assessed using the Medical Research Council scale (MRC; scores range from zero to 60, with higher scores indicating greater strength).^(23)^ Muscle weakness was defined as an MRC score < 48. Frailty was assessed using the modified frailty index (MFI, scores range from zero to 11, with higher scores indicating higher frailty).^(24)^ Cognition was assessed using the Mini Mental State Examination (MMSE, scores range from zero to 30, with higher scores indicating better cognition).^(25)^ Resilience was assessed using the Connor-Davidson resilience scale (scores range from zero to one hundred, with higher scores reflecting higher resilience).^(26)^ Anxiety and depression symptoms were assessed using the Hospital Anxiety and Depression Scale (HADS, scores on anxiety and depression scales range from zero to 21, with higher scores indicating worse symptoms).^(27)^ A cutoff > 7 for the HADS anxiety and depression subscales was used to define possible anxiety and depression. Symptoms of PTSD were assessed using the Impact Event Scale-6 (IES-6, scores range from zero to 24, with higher scores indicating worse symptoms);^(28)^ a cut off > 10 was used to define possible PTSD.

### 6-Minute Walk Test

The 6MWT was supervised by ratters not associated with care in accordance with international guidelines^(29)^ as part of the baseline post-ICU follow-up assessment (4 months after ICU discharge). Subjects were instructed to walk along a 30-meter corridor for 6 minutes and received encouragement during the test.

### Outcome

The main outcome was the variation in physical functional status based on the BI^(22)^ over the eight-month follow-up period (BI score at 8 months minus BI score at baseline). Subjects with BI variation ≥ +5 points were classified as having improved their physical functional status. Subjects who died during the follow-up were classified as not having improved their physical functional status. Investigators not associated with care and blinded to baseline variables performed both BI evaluations using structured telephone interviews.^(30)^

### Statistical analysis

Continuous variables are expressed as the median and interquartile range (IQR). Categorical variables are expressed as counts and percentages. The paired sample t-test or the Wilcoxon sign rank-test were used to compare 6MWT distances completed by study participants with expected values for heathy individuals (adjusted by gender, age, and body mass index - BMI),^(31)^ COPD subjects,^(32)^ and heart failure subjects.^(33)^ Stepwise multivariate linear regression was used to assess factors associated with the distance walked in the 6MWT. All variables with a p < 0.20 in the univariate analysis were included in the multivariate model using the forward selection procedure with stopping rules based on a cutoff of 0.05 for p-values. The association between the distance walked in the 6MWT and improvement in physical functional status over time was assessed through logistic regression. The accuracy of the 6MWT in predicting improvement in physical functional status was evaluated using actual and estimated values for the area under the Receiver Operating Characteristic curve (AUROC). An AUROC greater than 0.8 indicated good prediction performance, whereas an AUROC between 0.6 - 0.8 and lower than 0.6 indicated moderate and poor prediction performance, respectively. The significance level adopted was 5%. All analyses were performed with R software (R Development Core Team).^(34)^

## RESULTS

### Study population characteristics

**Figure 1:**
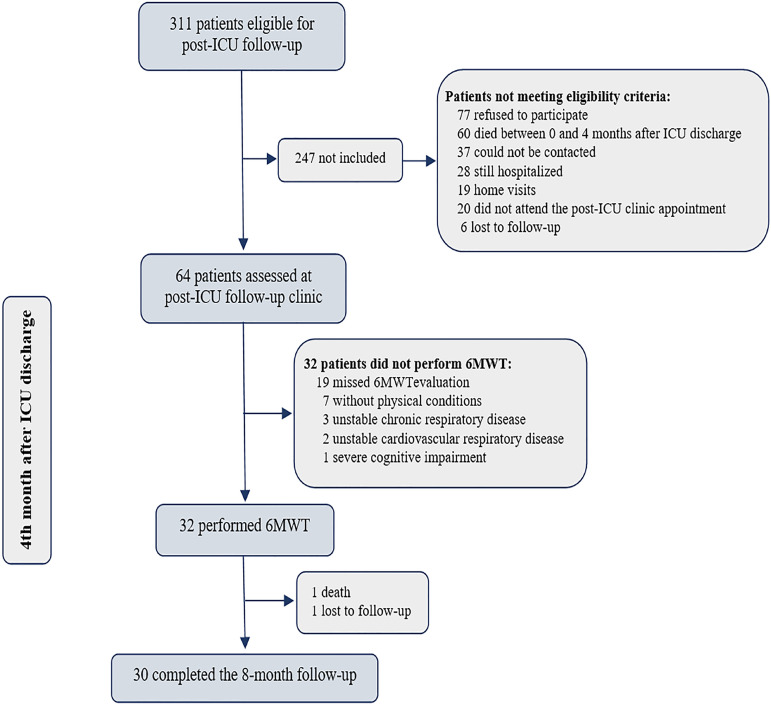
Participant flow diagram. ICU - intensive care unit; 6MWT - 6-Minute Walk Test.

From February 2017 to August 2018, 311 subjects were screened (Figure 1). Of these, 64 were assessed for eligibility. Eventually, 32 subjects were able to perform the 6MWT and were enrolled in the study. Of these subjects, 30 completed the protocol (one patient died, and one patient was lost to follow-up before the eight-month evaluation). Table 1 summarizes the characteristics of the study population. The median age was 58.5 years (IQR 37.5 - 67.5), and 56.2% of participants were women. Median education attainment was 11.0 years (IQR 9.8 - 16.0). Regarding critical illness, 71.9% of subjects were admitted to the ICU due to medical reasons and 22.1% due to surgery. The median risk of death at ICU admission was 18.6% (IQR 11.3 - 35.7). Sepsis or septic shock was present in 40.6% of subjects at the moment of ICU admission. The median number of organ dysfunctions during the ICU stay was 1.0 (IQR 0 - 2.3), and the median length of ICU stay was 7 days (IQR 4.0 - 11.0). At the moment of post-ICU outpatient assessment, 34.3% were functionally independent, 31.2% had high comorbidity, 77.4% had muscle weakness, 32.3% had possible anxiety, 16.1% had possible depression, and 22.6% had possible PTSD. The median MFI, MMSE, and Connor-Davidson Resilience Scale scores were 2.0 (IQR 0 - 3), 26.0 (IQR 23.0 - 29.0), and 81.0 (IQR 70.0 - 87.0), respectively.

**Table 1:** Patient characteristics

Characteristics	Values
Sociodemographic	
Age (years)	58.6 (37.5 - 67.5)
Female sex	18/32 (56.2)
Education attainment (years)	11.0 (9.8 - 16.0)
Higher education	11/32 (34.4)
Critical illness	
ICU admission type (%)	
Medical, emergency	23/32 (71.9)
Surgical, elective	5/32 (15.6)
Surgical, emergency	4/32 (12.5)
Risk of death at ICU admission* %	18.6 (14.2 - 33.7)
Severe sepsis or septic shock at ICU admission	13/32 (40.6)
Number of organ dysfunctions during ICU stay	1.0 (0 - 2.3)
Length of ICU stay	7.0 (4.0 - 11.0)
Health status at the moment of post-ICU outpatient assessment	
Physical functional status	
Independent	11/32 (34.3)
Mildly dependent	17/32 (53.1)
Moderately dependent	4/32 (12.5)
Severely dependent	0
Charlson Comorbidity Index	0 (0 - 2)
High comorbidity†	10/32 (31.2)
MRC strength score	58 (50.0 - 60.0)
Muscle weakness‡	24/32 (77.4)
Modified frailty index	2.0 (0 - 3.0)
MMSE score	26.0 (23.0 - 29.0)
Connor-Davidson resilience score	81.0 (70.0 - 87.0)
HADS anxiety score	6.0 (3.5 - 9.5)
Possible anxiety§	10/31 (32.3)
HADS depression score	4.0 (2.5 - 6.5)
Possible depression¶	5/31 (16.1)
IES-6 score	5.0 (2.0 - 8.5)
Possible PTSD||	7/31 (22.6)

ICU - intensive care unit; MRC - Medical Research Council; MMSE - Mini Mental State Examination; HADS - Hospital Anxiety and Depression Scale; IES-6 - Impact Event Scale-6; PTSD - posttraumatic stress disorder. * According to the Simplified Acute Physiology Score 3 or the Acute Physiology and Chronic Health Evaluation II score; † Charlson Comorbidity Index ≥ 2; ‡ Medical Research Council score < 48; § Hospital Anxiety and Depression Scale anxiety score > 7; ¶ Hospital Anxiety and Depression Scale depression score > 7; || Impact Event Scale-6 score >10. Results expressed as median (interquartile range) or n/total n (%).

### Results of the 6-Minute Walk Test

**Figure 2:**
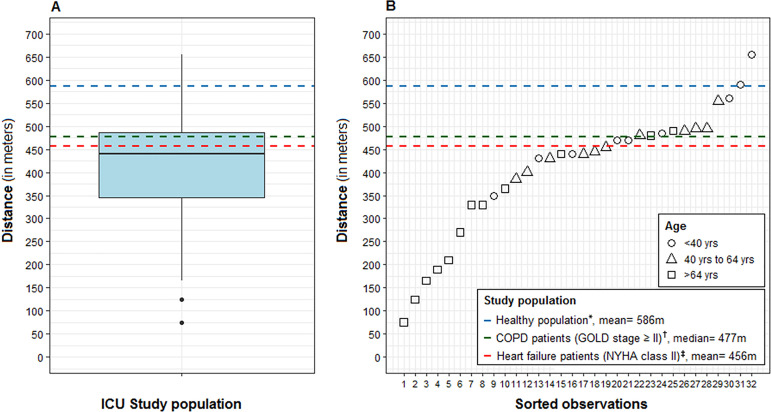
Distance walked in the 6-Minute Walk Test by intensive care unit survivors. (A) Summary of the distance walked in the 6-Minute Walk Test by intensive care unit survivors (box plot) compared with healthy individuals (blue dashed line), chronic obstructive pulmonary disease patients (green dashed line), and heart failure patients (red dashed line). (B) The sorted observations of the 6-Minute Walk Test distance among study subjects. * According to Britto et al.;^(31)^ † according to Rodrigues et al.;^(32)^ ‡ according to Oliveira et al.^(33)^ COPD - chronic obstructive pulmonary disease; GOLD - Global Initiative for Chronic Obstructive Lung Disease; NYHA - New York Heart Association.

Data regarding the distance walked in the 6MWT and the comparison across distinct populations are shown in figure 2. The mean distance walked was 405m (standard deviation of 135.9m). The mean 6MWT distance of ICU survivors was significantly lower than the predicted values for a healthy population considering sex, age, and BMI (405m *versus* 557m; p < 0.001). Intensive care unit subjects also walked lower distances than COPD subjects classified as Global Initiative for Obstructive Lung Disease (GOLD) stage ≥ II (median 455m *versus* 477m; p < 0.001) and heart failure subjects classified as New York Heart Association (NYHA) class II (mean 405m *versus* 456m; p = 0.02).

### Factors associated with 6-Minute Walk Test distance

Univariate and multivariate analyses of factors associated with distance walked in the 6MWT are shown in table 2. Upon multivariate analysis, older age (b-4.0; p < 0.001) and muscle weakness (b-99.7; p = 0.02) were independently associated with lower 6MWT distances.

**Table 2:** Factors associated with the distance covered in the six-minute walk test

Variables	Univariate analysis		Multivariate analysis*	
	ß (95%CI)	p value	ß (95%CI)	p value
Sociodemographic				
Age (years)	-4.7 (-6.91 - -2.64)	< 0.001	-4.0 (-6.1 - -1.9)	<0.001
Female sex	-37.0 (-136.6 - 62.5)	0.45	-	-
Education attainment (years)	7.5 (-2.1 - 17.2)	0.12	-	-
Higher education	24.2 (-80.4 - 128.8)	0.64	-	-
Critical illness				
ICU admission type (%)				
Medical, emergency	Reference	-	-	-
Surgical, elective	-6.8 (-146.1 - 132.5)	0.92	-	-
Surgical, emergency	73.4 (-79.5 - 226.4)	0.33	-	-
Risk of death at ICU admission per 1% increase	0.4 (-2.6 - 3.5)	0.77	-	-
Sepsis or septic shock at ICU admission	-35.2 (-135.9 - 65.4)	0.48	-	-
No. of organ dysfunctions during ICU stay	18.1 (-17.0 - 53.3)	0.30	-	-
Length of ICU stay (days)	-2.31 (-7.9 - 3.2)	0.40	-	-
Post-ICU clinic assessment				
Physical functional status				
Independent	Reference	-	-	-
Mildly dependent	115.0 (-31.5 -261.5)	0.13	-	-
Moderately dependent	191.5 (38.5 - 344.6)	0.01	-	-
Charlson comorbidity index per point	-17.0 (-48.0 - 13.9)	0.26	-	-
High comorbidity	-66.0 (-170.8 - 38.6)	0.20	-	-
MRC strength score per point	5.6 (1.19 - 10.1)	0.01	-	-
Muscle weakness	-133.8 (-240.5 - -27.1)	0.01	-99.7 (-188.5 - -10.9)	0.02
Modified frailty index per point	-26.2 (-53.4 - 0.9)	0.05	-	-
MMSE score per point	14.6 (5.5 - 23.6)	0.002	-	-
Connor-Davidson resilience score per point	0.61 (-3.1 - 4.3)	0.74	-	-
HADS anxiety score per point	2.2 (-8.1 - 12.6)	0.66	-	-
Possible anxiety	-2.3 (-101.3 - 96.5)	0.96	-	-
HADS depression score per point	5.2 (-6.2 - 16.6)	0.36	-	-
Possible depression	19.5 (-105.9 - 145.0)	0.75	-	-
IES-6 score per point	3.3 (-6.1 - 12.8)	0.47	-	-
Possible PTSD	59.7 (-48.5 - 167.9)	0.26	-	-

95% CI - 95% confidence interval; ICU - intensive care unit; MRC - Medical Research Council; MMSE - Mini Mental State Examination; HADS - Hospital Anxiety and Depression Scale; IES-6 - Impact Event Scale-6; PTSD - posttraumatic stress disorder. * The following variables were entered in the multivariate model: education attainment, physical functional status, muscle weakness, modified frailty index and Mini Mental State Examination score. We did not include the Medical Research Council strength score due to collinearity between this variable and muscle weakness. Only variables with a p value < 0.05 in the multivariate model are shown.

### Association between 6-Minute Walk Test distance and improvement in physical functional status

**Figure 3:**
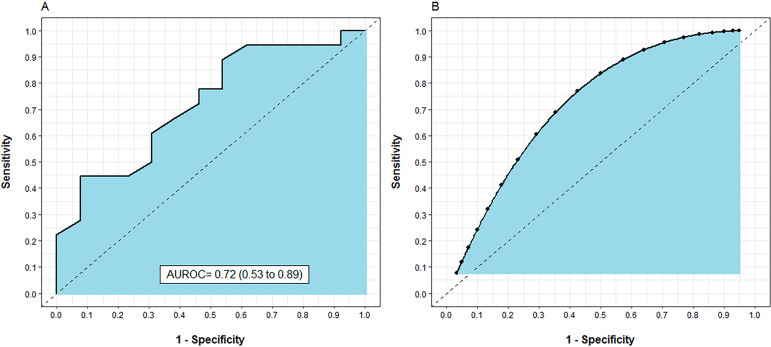
Actual and predicted area under the Receiver Operating Characteristic curve for the ability of the 6-Minute Walk Test to predict improvement in physical functional status over time. (A) The actual area under the Receiver Operating Characteristic (95% confidence interval). (B) The predicted area under the Receiver Operating Characteristic for the ability of the 6-Minute Walk Test to predict improvement in physical functional status over time. AUROC - area under the Receiver Operating Characteristic curve.

Eighteen subjects (58.0%) had their physical functional status improved during the follow-up period. The mean 6MWT distance covered by subjects with improved physical functional status was significantly higher than that of subjects whose functional status did not improve (451m *versus* 340m; p = 0.03). The odds ratio of each additional 10m of walking in the 6MWT for improvement of physical functional status was 1.07 (95% confidence interval - 95%CI 1.01 - 1.16). The analysis of the AUROC for the prediction of improvement in physical functional status showed moderate predictive accuracy for the 6MWT (AUROC 0.72; 95%CI 0.53 - 0.88) (Figure 3).

## DISCUSSION

In this prospective cohort study involving ICU survivors attending a post-ICU outpatient clinic four months after ICU discharge, the 6MWT was able to predict long-term physical improvement with moderate accuracy. Additionally, we found that older age and muscle weakness were associated with lower distances covered in the 6MWT four months after ICU discharge.

Nearly two-thirds of ICU survivors are affected by physical dysfunction, which is typically long lasting and associated with a reduced quality of life.^(35-37)^ Common causes of physical disability among ICU survivors include loss of muscle mass, joint dysfunction, chronic pain, and reduced tolerance to exercise, which are potentially treatable conditions. Our findings are consistent with previous studies demonstrating the low exercise capacity of ICU survivors as measured by the 6MWT.^(13-16,35,36)^ Notably, in our study, the mean distance walked in the 6MWT by ICU survivors was significantly lower than that covered by healthy individuals and subjects with chronic diseases that impact quality of life (i.e., COPD and heart failure).^(38,39)^ In ICU survivors, the 6MWT is usually employed as a tool to determine physical disability rather than as a prognostic marker of physical improvement over time.^(13-16,35,36)^ In our study, the distance walked in the 6MWT showed promising results as a predictor of improvement in physical function over an eight-month period. Additionally, we found that older age and muscle weakness were associated with lower distances covered in the 6MWT four months after ICU discharge. Age is associated with frailty, comorbidities, and muscle function impairment-the main factors related to reduced walking ability. These results have clinical applicability, given the increasing interest in assessing physical disability in post-ICU care^(40)^ and the need to improve long-term outcomes of survivors of critical illness.^(1-3,41)^ The efficacy of the current post-ICU follow-up models for the improvement of physical disability has been questioned,^(42,43)^ and the use of personalized rehabilitation strategies based on individual risk factors for long-term physical disability has been proposed as a promising approach for ICU survivors.^(1-3,42)^ In this sense, the use of prognostic tools may contribute to a more efficient allocation of resources aimed at rehabilitating subjects who would benefit the most from this kind of intervention.

The strengths of our study include its prospective design, the start of follow-up after ICU discharge, during routine outpatient rehabilitation care, and the focus on the predictive accuracy of the 6MWT for long-term physical functional status improvement. Nevertheless, some limitations must be considered. First, our sample was small and, therefore, may not be representative of all ICU survivors, given the peculiarities of ICU subjects in specific contexts, such as trauma, surgery, and sepsis. Second, our study is susceptible to biases inherent to observational studies (i.e., confounding and selection and assessment bias). However, the possibility of systematic errors was minimized by adequate measurement of variables and outcomes with previously defined objective criteria, the use of standardized data collection, and follow-up performed by a research team that was not involved in patient care.

## CONCLUSION

The distance covered in the 6-Minute Walk Test appears useful for assessing the long-term physical functional status recovery among intensive care unit survivors.
